# RBS1, an RNA Binding Protein, Interacts with SPIN1 and Is Involved in Flowering Time Control in Rice

**DOI:** 10.1371/journal.pone.0087258

**Published:** 2014-01-30

**Authors:** Yuhui Cai, Miguel E. Vega-Sánchez, Chan Ho Park, Maria Bellizzi, Zejian Guo, Guo-Liang Wang

**Affiliations:** 1 State Key Laboratory for Biology of Plant Diseases and Insect Pests, Institute of Plant Protection, Chinese Academy of Agricultural Sciences, Beijing, China; 2 College of Agronomy and Biotechnology, China Agricultural University, Beijing, China; 3 Department of Plant Pathology, Ohio State University, Columbus, Ohio, United States of America; National Taiwan University, Taiwan

## Abstract

The rice U-box/ARM E3 ubiquitin ligase SPL11 negatively regulates programmed cell death (PCD) and disease resistance, and controls flowering time through interacting with the novel RNA/DNA binding KH domain protein SPIN1. Overexpression of *Spin1* causes late flowering in transgenic rice under short-day (SD) and long-day (LD) conditions. In this study, we characterized the function of the RNA-binding and SPIN1-interacting 1 (RBS1) protein in flowering time regulation. *Rbs1*was identified in a yeast-two-hybrid screen using the full-length *Spin1* cDNA as a bait and encodes an RNA binding protein with three RNA recognition motifs. The protein binds RNA *in vitro* and interacts with SPIN1 in the nucleus. *Rbs1* overexpression causes delayed flowering under SD and LD conditions in rice. Expression analyses of flowering marker genes show that *Rbs1* overexpression represses the expression of *Hd3a* under SD and LD conditions. *Rbs1* is upregulated in both *Spin1* overexpression plants and in the *spl11* mutant. Interestingly, *Spin1* expression is increased but *Spl11* expression is repressed in the *Rbs1* overexpression plants. Western blot analysis revealed that the SPIN1 protein level is increased in the *Rbs1* overexpression plants and that the RBS1 protein level is also up-regulated in the *Spin1* overexpression plants. These results suggest that RBS1 is a new negative regulator of flowering time that itself is positively regulated by SPIN1 but negatively regulated by SPL11 in rice.

## Introduction

Flowering time is controlled by endogenous and environmental signals. In Arabidopsis, a long day (LD) plant, extensive genetic analyses have identified four flowering pathways: photoperiod, autonomous, vernalization, and gibberellin induced pathways, as well as a series of signaling molecules [1], [2], [3], [4], [5]. Among them, the FLOWERING LOCUS T (FT) protein is the florigen signal, which moves from stem vascular tissue to the shoot apical meristem (SAM) to promote flowering [6]. CONSTANS (CO) is a key regulator of the photoperiod pathway. CO is a positive regulator of FT and is induced under LD conditions [7], [8].

Rice, a short day (SD) plant, has a similar molecular mechanism to that of Arabidopsis. Rice heading date 3a (Hd3a) and heading date 1 (Hd1) are the orthologs of FT and CO, respectively [9], [10]. Hd1 promotes flowering under SD conditions but inhibits Hd3a under LD conditions. The early heading date 1 (Ehd1) protein positively regulates the expression of Hd3a, influencing the flowering time in rice independent of Hd1. This pathway only exists in rice as there is no Ehd1 ortholog in *Arabidopsis* [11]. Previous research has shown that Hd3a and its homologue RICE FLOWERING LOCUS T1 (RFT1), act as rice florigens that activate the expression of OsMADS15 [12], [13]. Recently, it has been shown that the rice florigen protein Hd3a interacts with 14-3-3 proteins in the apical cells of shoots, yielding a complex that translocates to the nucleus and binds to the OsFD1 transcription factor to regulate flowering time [14].

RNA binding proteins are involved in the synthesis, processing, transportation, translation, and degradation of various types of RNA molecules in the cell [15]. In *Arabidopsis*, there are 196 genes encoding RNA recognition motif (RRM)- containing proteins [16]. The RNA binding protein FCA has two RRMs and promotes flowering under LD condition [17]. Another RNA biding protein gene, *FLK*, regulates the autonomous flowering pathway via FLC in Arabidopsis [18]. More recently, another RRM-containing protein, LIF2, was identified in *Arabidopsis* and shown to be involved in flowering time control and cell fate [19]. In rice, there are at least 221 RNA binding proteins [20]. Among them, 31 contain the RRM RNA binding motif. The function of the RRM RNA binding proteins in rice is unknown.

We previously demonstrated that Spotted Leaf 11 (SPL11), a functional U-box/ARM E3 ligase, is involved in the regulation of programmed cell death (PCD), defense and flowering time in rice [21], [22], [23]. In a yeast-two hybrid screen using SPL11 as the bait, we identified the SPL11 Interact Protein 1, SPIN1, which is a KH domain RNA binding protein. SPL11 monoubiquitinates SPIN1 in vitro and interacts with SPIN1 in the nucleus [22]. Overexpression of *Spin1* in transgenic rice causes late flowering under SD and LD conditions [22]. In order to identify proteins that interact with SPIN1, we performed a yeast two-hybrid screen using *Spin1* full-length cDNA as the bait. We identified a novel RRM-containing protein that we have named RNA-binding and SPIN1-interacting 1 (RBS1). RBS1 interacts with SPIN1 *in vitro* and *in vivo* and binds RNA in vitro. Overexpression of *Rbs1* in transgenic rice leads to late flowering under both SD and LD conditions. These results revealed that RBS1 plays a negative role in rice flowering and is a new component in the SPL11-mediated signaling pathway.

## Materials and Methods

### Measurement of the Flowering Time Under SD and LD Conditions

Rice (*Oryza sativa*) seeds of mutant, transgenic and wild-type plants were sterilized and germinated in half-strength Murashige and Skoog medium for 7 d, and then transferred to a pot with sterilized soil in a growth chamber with the same conditions as described by Vega-Sánchez et al. (2008). *Rbs1-ox* plants were generated in the cv. Nipponbare background, while the knockout mutant was obtained from the Postech collection in Korea (cv. Dongjin). For flowering time measurements, plants were grown either in 10/14 h light/dark for SD or 14/10 h light/dark for LD. Rice flowering time was measured in days from germination until emergence of the first panicle. For diurnal expression analyses, young leaves were harvested from wild-type Nipponbare, *Rbs1* overexpression lines, Dongjing and *rbs1* plants of 50 day-old (SD) or 60 day-old (LD) plants at 4 h intervals for a total of 24 h.

### Yeast Two-hybrid Screen

The ProQuest yeast two-hybrid system (Invitrogen) was used to screen for SPIN1-interacting proteins following the manufacturer’s protocol. A full-length *Spin1* cDNA was used as the bait by cloning it into the pDBleu vector (*Sal*I and *Not*I sites). A rice cDNA library available into the pPC86 vector was used as the prey (22). Putative interacting candidates were identified by sequencing of the inserts in pPC86 at the Plant-Microbe Genomics Facility (PMGF) at the Ohio State University.

### Subcellular Localization Experiments

The *DsRed-Rbs1* translational fusion was obtained by cloning an *Rbs1* coding sequence PCR fragment containing the *Bgl*II and *Sal*I sites into the pGDR vector to make pGRbs1-Red. The *GFP-Spin1* translational fusion was made in the pGDG vector (pGSpin1-GFP) as described previously [22]. The pGRbs1-Red and pGSpin1-GFP constructs were transformed into *Agrobacterium tumefasciens* strain GV3101 and used to agroinfiltrate 4 week-old *N. benthamiana* plants as described previously [24]. Two days after infiltration, infiltrated leaves were observed under a fluorescent microscope to detect the localization of DsRed-RBS1 and/or GFP-SPIN1 fusion proteins. For subcellular localization in rice cells, the same constructs used for agroinfiltration were used to transform rice seedling protoplasts. The rice protoplast isolation and PEG-mediated transformation, fluorescence microscope settings and filters were as described [25].

### Reverse Transcription (RT)-PCR Analyses

Total RNA was extracted using Trizol reagent (Invitrogen) following the manufacturer’s protocol. RNA was treated with DNAse1 (Invitrogen) and 1.5 µg was used for first strand cDNA synthesis with the reverse transcription system from Promega, following the kit’s instructions. Real-time quantitative RT-PCR (qPCR) was performed in a final volume of 50 µL, including 25 µL iQ SYBR Green Supermix (Biorad), 2 µL of the diluted first-strand cDNA as templates and 0.2 µM of each primer. The reactions were carried out with the Biorad IQ5 system in the following program: 94°C for 4 min, 40 cycles of 95°C for 5 s, and 58°C for 20 s, 72°C for 30 s. Every experiment was repeated at least three times. The primers of *Hd1*, *Hd3a*, *Ubq*, *Rbs1*, *Spin1* and *Spl11* are listed in Table S1. The level of ubiquitin (UBQ) expression was used to normalize the expression ratio of each gene.

### RNA Binding Assay

The *Rbs1* coding sequence was cloned by PCR into the *Hin*dIII and *Xho*I sites of pET28a to obtain pET-Rbs1. As a negative control, unrelated protein Avr-Pita was cloned into *Bam*HI and *Sal*I site of the same vector and designated as pET-AvrPita. The6XHIS-RBS1 or 6XHIS-Avr-Pita protein was induced in *E. coli* and purified using HIS-Select® Nickel Affinity Gel (Sigma) according to manufacturer’s instruction. Either purified 6XHIS-RBS1 or 6XHIS-Avr-Pita was incubated with beads containing polyuridylic, and polyguanylic ribohomopolymers as well as calf thymus single- and double stranded DNA purchased from Sigma. Incubation was done in 500 µl of buffer KHN (150 mM KCl, 20 mM HEPES pH7.9, 0.01% NP-40, complete protease inhibitors) for 10 min under rotation. Beads were then washed in KHN buffer 5 times and proteins retained in the beads were identified by Immunoblotting using anti-HIS antibody (Thermo Scientific).

### Constructs

In general, constructs were generated by PCR amplification of the target gene using primers containing appropriate restriction enzyme sites and *Spin1* or *Rbs1* cDNA template, followed by ligation into a desired vector. For the BiFC assay, the *Rbs1* and *Spin1* constructs in the pA7-NYFP and pA7-CYFP vectors were constructed following the procedure by Chen et al. (2006). The *Rbs1* overexpression construct was made into the Gateway (Invitrogen)-compatible vectors Ubix.nc1300.ntap.gck, following the Gateway cloning protocols. Two artificial miRNA constructs were made that targeted the 21 bp of *Rbs1*’s 3'UTR (gatggatttgttaacttatga, 50 bp after the stop code), which was obtained from the WMD3 website (http://wmd3.weigelworld.org/cgi-bin/webapp.cgi) [26]. The first construct has one mismatch and the second construct has two mismatches against the target site.

### BiFC Detection of the Interact between SPIN1 and RBS1

Fluorescence microscopy analyses were done by transfection of rice protoplasts with various constructs as described [25].

#### In vivo Co-IP assays

For *in vivo* Co-IP assay, *Agrobacterium* strain GV3101 carrying expression vectors of *RFP:RBS1*and *SPIN1:HA*were agroinfiltrated *N. benthamiana* leaves to express the proteins in vivo. Two days after agroinfiltration, *N. benthamiana* leaf tissues were harvested, and total proteins were extracted with a native buffer including 50mM K+-HEPES (pH 7.4), 110mM KOAc, 2mM MgCl_2_, 0.1%Tween-20, 0.2% Triton, and plant protease inhibitor. A 15-µl volume of anti-HA agarose suspension (Sigma) was added to the protein samples loaded in the column, and the mixtures were kept at 4°C with head-to-tail shakingovernight. The samples were washed five times using 1X IP buffer following the manufacturer’s instruction (Sigma). After 50 µl of 1X SDS sample loading buffer was added to each column, samples were heated to 95°C for 5 min. Twenty microliters of each sample was loaded to the protein gel for immunoblot analysis using anti-HA (Sigma) and anti-RFP (BGI, China) antibodies.

### Western Blot

The protein extraction and protein gel blot assay were done as described [27]. Anti-HA tag antibody (Roche), Anti-RBS1 antibody(Beijing Protein Innovation, China), Anti-SPIN1 antibody(Beijing Protein Innovation, China) and Anti-peroxidase (PAP) antibody (Sigma-Aldrich) were used to detect these proteins. Chemiluminescene was detected by the ChemiDoc XRS system (Bio-Rad).

## Results

### RBS1 Interacts with SPIN1 in Yeast and in Rice Protoplasts and Encodes a Heterogeneous Nuclear Ribonucleoprotein R-type Protein

To identify proteins that interact with the RNA binding protein SPIN1, a yeast two-hybrid screen was performed using the full-length *Spin1* cDNA as the bait and a rice cDNA library as the prey. Approximately half a million-yeast colonies were screened and 140 putative positive clones were selected for validation after the first round of screening. Only clones #69-2 and 72 were able to reproduce the interaction based on yeast growth on different selection media and appearance of blue colonies in an X-GAL assay (Figure S1). Sequencing analysis revealed that both 69-2 and 72 clones contained the same cDNA insert.

**Figure 1:**
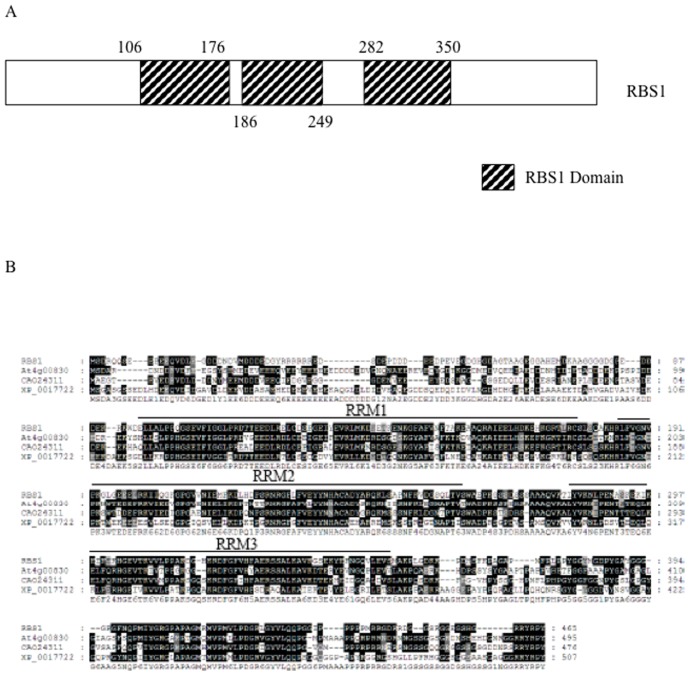
RBS1 is an RNA binding protein with three RNA Recognition Motifs (RRM). (A) Schematic representation of the RBS1 protein (B) Multiple sequence alignment of RBS1 and its putative plant orthologs. At4g00830, from *Arabidopsis thaliana*; CAO24311 from grape (*Vitis vinifera*); XP_0017722 from the moss *Physcomitrella patens* subsp. *patens*. The alignment was performed with ClustalX and shading of amino acid residues with Genedoc.

BLAST searches showed that the insert corresponded to the full-length cDNA of the rice gene LOC_*Os11g14430*. The deduced amino acid sequence of *Os11g14430* encodes a protein of 465 residues with a predicted molecular weight of 51.2 KDa and a theoretical pI of 5.15. Searches in various protein databases, including GenBank and Pfam, using the deduced amino acid sequence revealed that the candidate SPIN1 interactor was a putative RNA binding protein containing three RRMs (Figure 1A). The candidate gene was then named *Rbs1*, for RNA-binding and SPIN1-interacting 1. The RRM is one of the most common domains found in heterogeneous nuclear ribonucleoproteins (hnRNP) [16]. Indeed, the RRM motifs in RBS1 were most similar to metazoan Apobec-1 complementation factor 1 (ACF1), a type of hnRNP R protein involved in apolipoprotein B mRNA editing [28]. The homology of RBS1 to ACF1 was limited to the RRM region with 36% identity and 53% similarity. Several plant RBS1-like proteins were identified in *Arabidopsis*, grape (*Vitis vinifera*) and the moss *Physcomitrella patens* subsp. *patens* (Figure 1B). So far, only the function of the *Arabidopsis* closest relative of RBS1 has been determined; it was shown that this protein, known as LIF2, interacts with the polycomb repressive complex protein LHP1 and is involved in flowering time and flower organ development [18].

### RBS1 Co-localizes and Interacts with SPIN1 in the Nucleus

**Figure 2:**
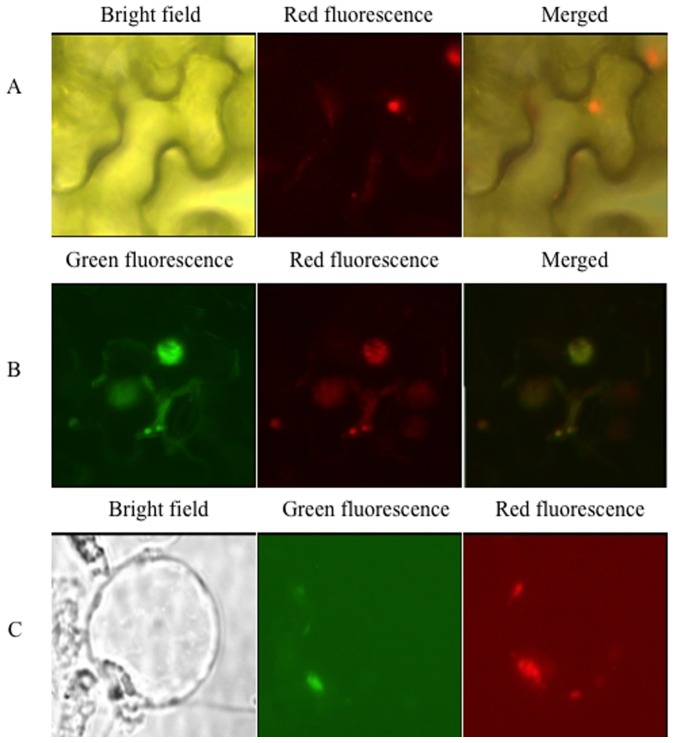
Subcellular localization of RBS1 in *N. benthamiana* and rice. (A) DsRed-RBS1 localization in the nucleus of *N. benthamiana* (B) GFP-SPIN1 and DsRed-RBS1 co-localization in the nucleus *of N. benthamiana* (C) GFP-SPIN1 and DsRed-RBS1 co-localization in the nucleus of rice protoplasts.

We have previously shown that SPIN1 is localized in the nucleus [22]. To determine the subcellular localization of RBS1, we expressed the fusion protein DsRed-RBS1 in *Nicotiana benthamiana*. Red fluorescence was observed in the nuclear region in the infiltrated cells (Figure 2A), indicating the nuclear localization of RBS1. We then co-expressed DsRed-RBS1 and GFP-SPIN1 in *N. benthamiana* and in rice protoplasts, separately. As shown in Figure 2B and 2C, SPIN1 and RBS1 co-localized in the nucleus of both *N. benthamiana* and rice cells.

**Figure 3:**
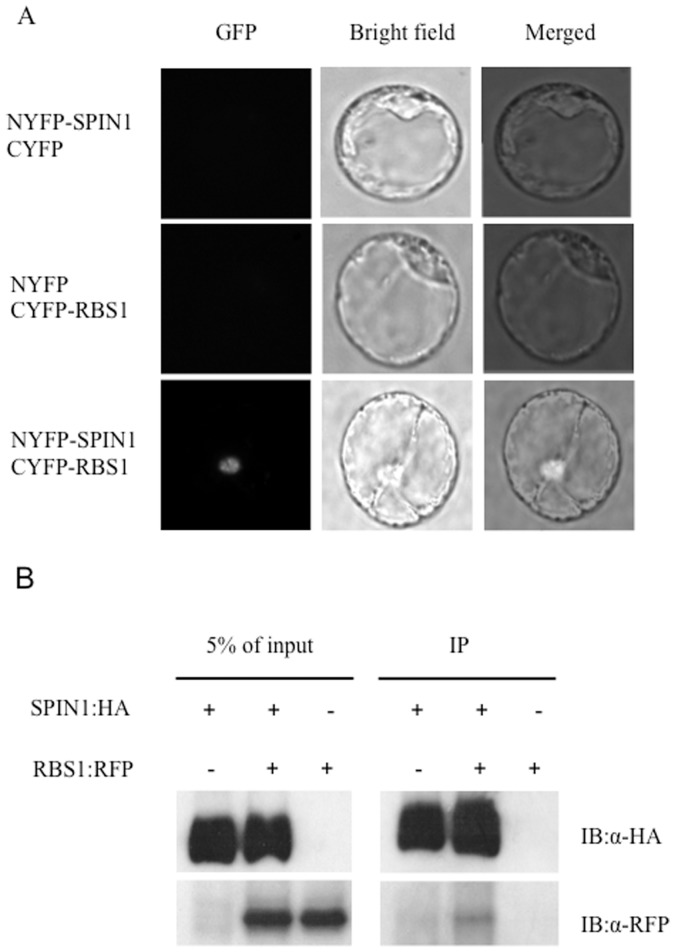
Interaction between SPIN1 and RBS1 *in planta*. (A) BiFC detection of the SPIN1–RBS1 interaction in rice protoplasts. (B) Co-IP of RBS1:RFP with SPIN1:HA in *N. benthamiana.*

To confirm the Y2H result, we used the bimolecular fluorescence complementation (BiFC) technique to test the interaction between SPIN1 and RBS1 in rice protoplasts [25], [28]. No fluorescence signal was detected when NYFP-SPIN1+ CYFP or NYFP+CYFP-RBS1 (Figure 3A) were expressed in rice protoplasts, respectively. However, fluorescence was fully reconstituted in the nucleus only when full-length SPIN1 and RBS1 proteins were co-expressed in rice protoplasts as NYFP and CYFP fusions, respectively (Figure 3A), indicating that these proteins interact *in vivo* and that the interaction occurs in the nucleus. To further confirm the interaction *in vivo,* we performed a co-IP experiment by expressing SPIN-HA and RBS1-RFP fusion proteins in *N. benthamiana* by agroinfiltration. As can be seen in figure 3B, RBS1-RFP co-immunoprecipitated with SPIN-HA.

### RBS1 has Nucleic Acids Binding Activity *in vitro*

**Figure 4:**
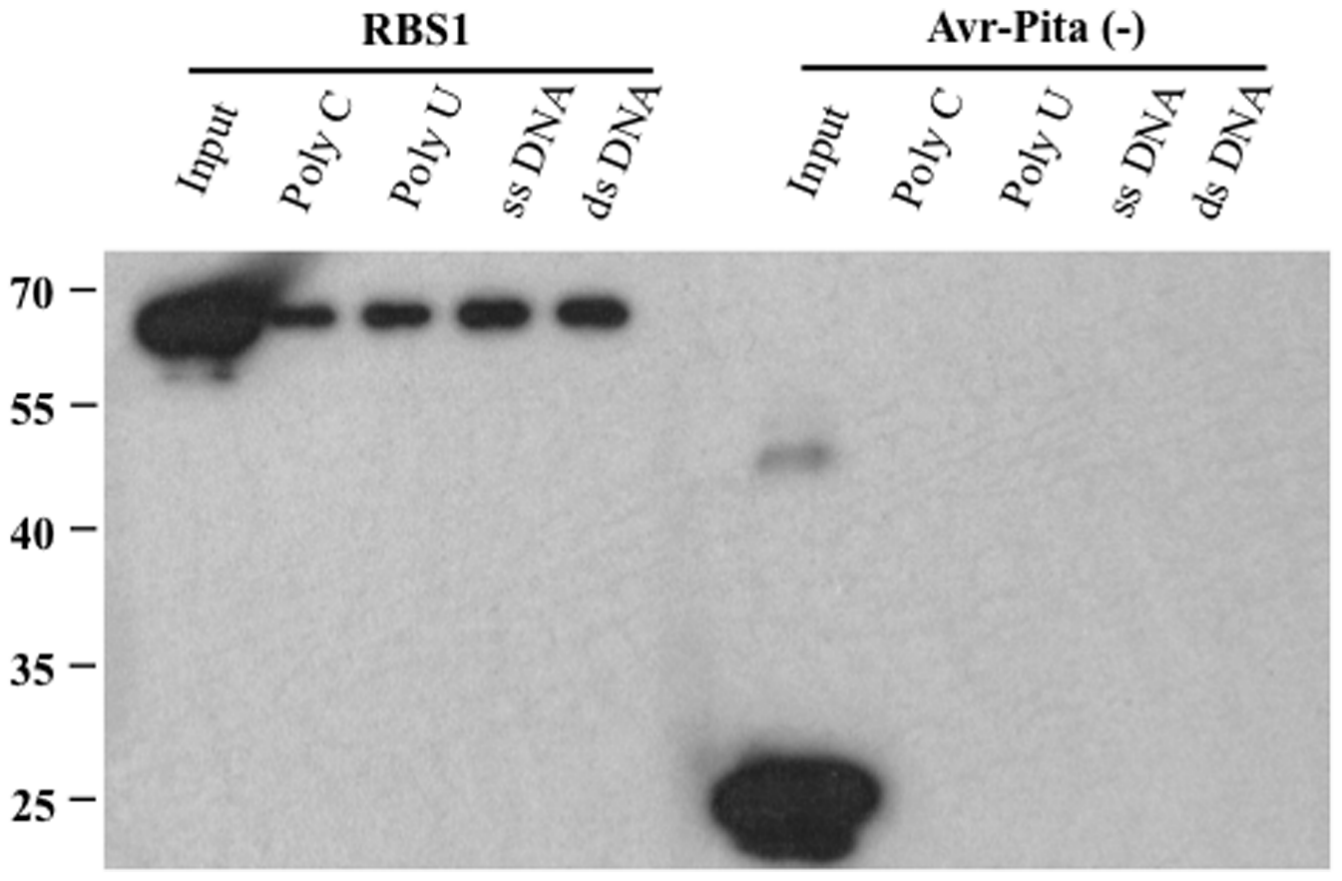
RNA and DNA binding assay for RBS1. *E. coli-*purified 6xHis-RBS1 protein was incubated with ribohomopolymers, ssDNA and dsDNA to test for *in vitro* nucleic acid binding activity. An unrelated protein, 6xHis-Avr-Pita was used as a negative control. Proteins were detected by western blot using an Anti-His antibody.

Our bioinformatic analyses suggested that RBS1 is a putative RNA binding protein. To confirm the *in silico* data, we performed an *in vitro* RNA binding assay. 6xHis-RBS1 protein was purified from *E. coli* and incubated with ribohomopolymer beads containing poly U and poly C RNA molecules, as well as calf thymus single and double-stranded DNA. Detection of proteins bound to nucleic acids was performed by western blot analysis using an anti-His antibody after SDS-PAGE and blotting. The assay showed that 6xHis-RBS1 bound to both RNA and DNA molecules *in vitro* (Figure 4). The unrelated avr-Pita protein was used as negative control in the same RNA and DNA binding experiments (Figure 4).

### *Rbs1* Overexpression Causes Late Flowering Under LD and SD Conditions

To analyze the function of *Rbs1*, we obtained the *rbs1* T-DNA mutant, PFG_4A-01883.R, from the rice T-DNA insertion sequence database [29]. In the mutant, the T-DNA inserted in the fourth intron of the *Rbs1* locus. The genotype was confirmed by PCR using *Rbs1* and T-DNA specific primers (Figure S2A and B). Real-time PCR analysis revealed that the transcription of RBS1 was completely abolished (Figure S2C). However, no significant difference in flowering time in LD and SD was found between the *rbs1* knockout mutant and the wild type Dongjin (Figure S2D). The *Rbs1* silencing transgenic lines produced by the artificial microRNA technique showed similar result (data not shown).

**Figure 5:**
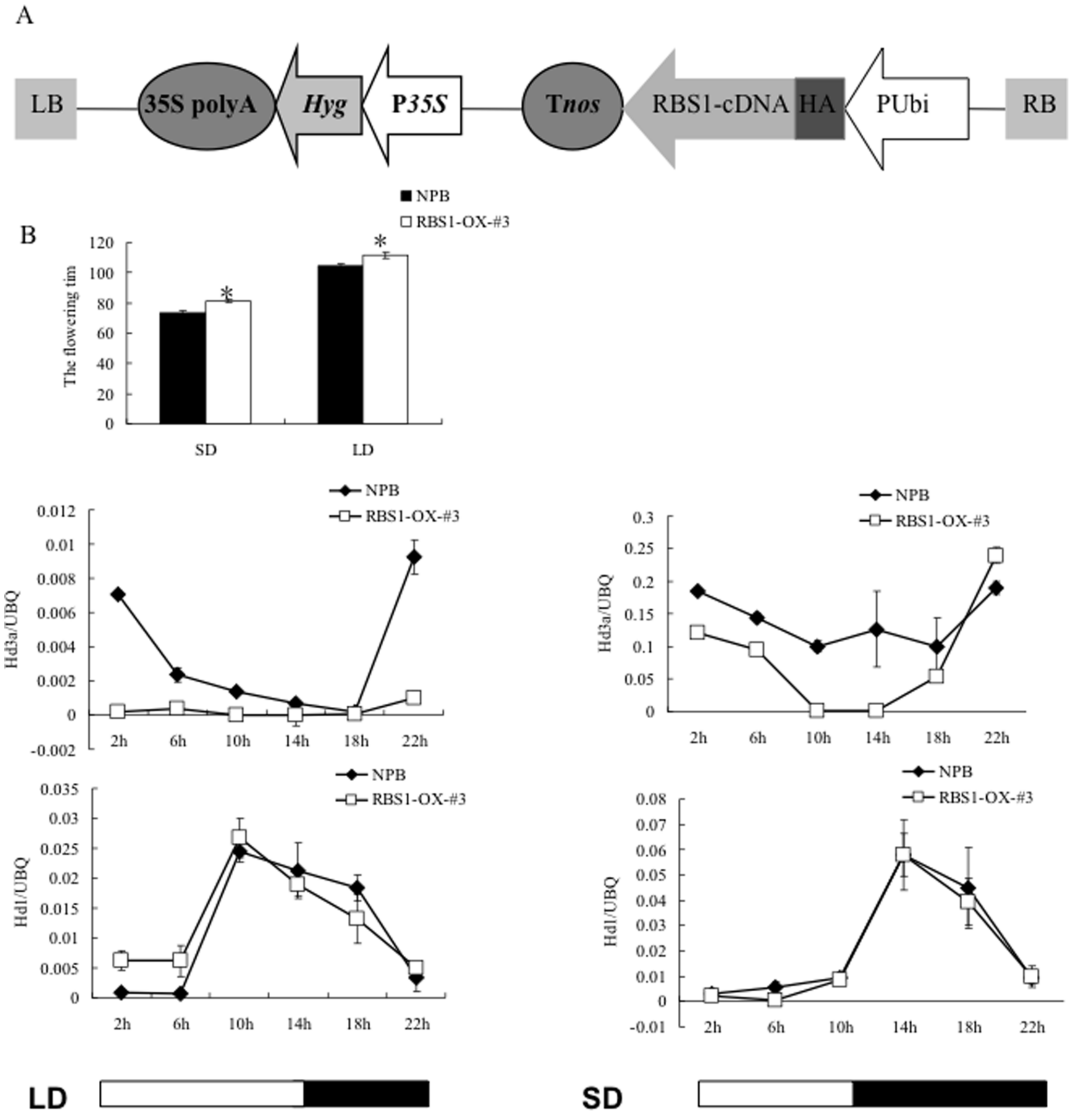
Flowering time of the *Rbs1* overexpression plants and the expression pattern of flowering marker genes under SD and LD conditions. (A) Schematic diagram of the *Rbs1* overexpression construct. The *Rbs1* cDNA sequence fused with the HA tag is placed after the maize ubiquitin promoter (B) Flowering time (days) of the *Rbs1* overexpression plants and wild type Nipponbare plants (NPB) was measured under SD and LD conditions (C) Real-time PCR analysis of the expression of *Hd3a* and *Hd1* in the *Rbs1* overexpression plants and wild type Nipponbare plants under SD and LD conditions.

We also constructed an *Rbs1* overexpression vector in which the *Rbs1* full-length cDNA was fused with an HA tag under the control of the maize ubiquitin (Ubi) promoter (Figure 5A). Through *Agrobacterium*-mediated transformation, we obtained more than twenty *Rbs1* overexpression transgenic lines. Among them, two T3 homozygous lines with high expression of the transgene, RBS1-OX-3 and -11, were used for the flowering time and gene expression analyses. To confirm the overexpression of *Rbs1* in the transgenic lines, we detected the RBS1 protein in the wild type and overexpression transgenic plants using both anti-RBS1 and anti-HA antibodies. Western blot analysis showed that the RBS1 protein level was high in both transgenic lines (Figure S3). On the contrary, no RBS1 band was detected in the wild type control plants.

In a previous study we showed that the *Spin1* overexpression plants flower late under SD and LD conditions and that the *spl11* mutant plants flower late under LD conditions. To evaluate whether *Rbs1* functions in flowering time, we grew the *Rbs1* overexpression and wild type Nipponbare plants under SD and LD conditions. Under both conditions, the *Rbs1* overexpression plants flowered one week later than the wild type plants (Figure 5B).

To gain insights into the *Rbs1*-mediated flowering mechanism, we detected the diurnal expression of the flowering marker genes *Hd3a* and *Hd1* in the *Rbs1* overexpression and the wild type plants using real-time PCR. Under SD and LD conditions, the expression of *Hd3a* was repressed in the *Rbs1* overexpression plants at most of the time points (Figure 5C), which is consistent with their late flowering phenotype and the previous report that *Hd3a*’s transcript is highly correlated with the flowering induction in the *Spin1* overexpression lines [22] and other genotypes [30]. No significant change in *Hd1* expression was observed between wild type NPB and *Rbs1-Ox* plants (Figure 5C). These results suggested that RBS1 may regulate the expression of *Hd3a* in SD to cause late flowering in rice.

### RBS1 Negatively Regulates *Spl11* Expression but Positively Regulates *Spin1* Expression

**Figure 6:**
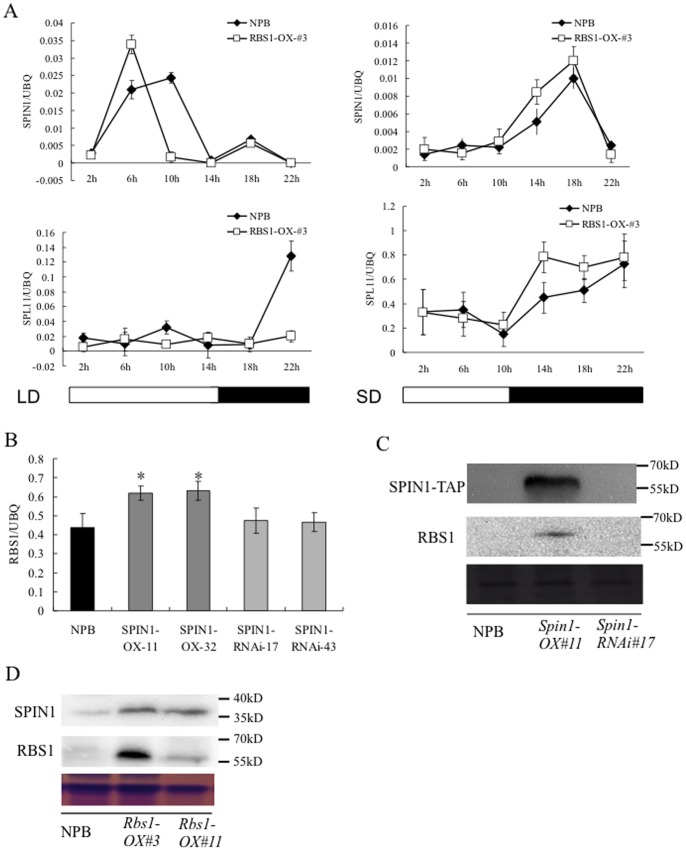
Gene expression and protein accumulation patterns of *Rbs1, Spin1* and *Spl11* inthe *Rbs1 and Spin1* overexpression plants. **A.** Q-PCR analysis of the expression pattern of *Spin1* and *Spl11* in the *Rbs1* overexpression plants under LD and SD conditions. B. Expression of *Rbs1* in the *Spin1* overexpression and RNAi plants. C. Western blot detection of RBS1 and SPIN1 proteins in the *Spin1* overexpression and RNAi plants. RBS1 was detected using a native antibody. The SPIN1-TAP fusion protein was detected in the *Spin1*-OX plants using the PAP antibody. D. Western blot detection of RBS1 and SPIN1 proteins in the *Rbs1* overexpression lines; RBS1 and SPIN1 native antibodies were used in the immunoblot detection.

Since SPIN1 interacts with SPL11 [22], we tested whether RBS1 also interacts with SPL11. No interaction between RBS1 and SPL11 was detected in yeast (data not shown). To determine the relationship among RBS1 and SPIN1 and SPL11 at the transcriptional level, we investigated the expression pattern of *Spin1* and *Spl11* in the *Rbs1* overexpression plants. Real-time PCR analysis showed that the expression of *Spin1* in the *Rbs1* overexpression plants was higher than the wild type at 6 h after dawn but lower at 10 h in LD (Figure 6A, left upper panel). At midnight, the expression of *Spl11* was repressed in the *Rbs1* overexpression plants (Figure 6A, left lower panel). However, the expression and *Spin1* and *Spl11* in the *Rbs1* overexpression plants in SD was not significantly different although both genes had higher expression at 14 h compared with that in wild type plants (Figure 6A, right upper and lower panels). We also detected the expression of *Rbs1* in the *Spin1* overexpression and RNAi plants and *spl11* mutant plants. The expression of *Rbs1* in the *Spin1* overexpression plants was higher than in the wild type plants, but did not vary significantly in *Spin1* RNAi plants (Figure 6B, Figure S4A). *Rbs1* expression was upregulated in the *spl11* mutant in the cultivar IR68 background at the old leaf stage when lesion mimics were present [31], correlating with the accumulation of many lesion mimics (Figure S4B). The upregulation of *Rbs1* in the *spl11* plants was also confirmed in the IR64 background (Figure S4C).

Furthermore, we determined the protein levels of RBS1 and SPIN1 in the *Rbs1* and *Spin1* overexpression plants using anti-RBS1 and anti-SPIN1 antibodies. Western blot analysis showed that enhanced accumulation of the RBS1 protein was detected in the *Spin1* overexpression plants (Figure 6C) and that the accumulation of the SPIN1 protein was higher in the *Rbs1* overexpression plants (Figure 6D). We also detected that the RBS1 protein level was increased in the *spl11* mutant (Figure S4D), which is consistent with the expression data mentioned above. These results suggest that there is a positive relationship between RBS1 and SPIN1 but a negative relationship between SPL11 and RBS1.

## Discussion

We identified RBS1 as a novel RNA binding protein of the hnRNP-R type in rice and showed that it interacts with SPIN1 in both yeast and rice protoplasts. To assess the functional relationship between *Rbs1* and *Spin1* in flowering time regulation in rice, we identified an *Rbs1* T-DNA insertion mutant and generated both overexpression and artificial microRNA transgenic plants of the *Rbs1* gene. Similar to the T-DNA mutant and the *Spin1* RNAi plants [22], the *Rbs1* artificial microRNA plants did not show any difference in flowering time compared with the wild type plants in both LD and SD conditions (Data not shown). However, like the *Spin1* overexpression plants [22], the *Rbs1* overexpression plants show late flowering under SD and LD conditions. Expression analysis showed that *Rbs1* overexpression leads to the suppression of both *Hd3a*, the rice florigen [12]. Our results clearly demonstrate that RBS1 is a flowering suppressor that interacts with SPIN1, and this interaction could lead to regulate the expression of flowering genes in rice.

Genome analysis showed that there are 196 RRM-containing proteins in *Arabidopsis* and 221 proteins in rice [16], [20]. In rice, LOC_*Os10g06130* also encodes an RRM-containing protein, which has 88% protein sequence similarity with RBS1. The expression of this gene is not affected in the *Rbs1* overexpression and artificial microRNA plants (data not shown). This gene may have redundantfunction with *Rbs1* in rice flowering, which might explain our observationthat the *rbs1* knockout mutant and artificial microRNA plants did not show any significant flowering time change under SD and LD conditions.

In the *Rbs1* overexpression plants, the expression of *Spin1* is upregulated, which correlates with enhanced SPIN1 protein accumulation. We have previously shown that the *Spin1* overexpression plants flower later than the wild type Nipponbare plants under SD and LD conditions [22]. In SD conditions, the expression of *Hd1* is reduced in the *Spin1* overexpression plants, indicating that SPIN1 influences flowering time via the Hd1 pathway in flowering-promoting conditions. However, in LD conditions, the *Hd1* expression, is the same in Nipponbare and *Spin1* overexpression plants [22], suggesting that SPIN1 may target an unknown factor to regulate *Hd3a* under LD conditions. By contrast, in this study we found that the expression of *Hd1* is similar between the *Rbs1* overexpression and wild type Nipponbare plants under both LD and SD conditions (Figure 5C). Interestingly, in the *Rbs1* overexpression plants, the mRNA level of *Hd3a* is suppressed under both SD and LD conditions. These data suggest that RBS1 may function to regulate flowering in rice via the suppression of Hd3a-mediated mechanisms.

Our previous study revealed the negative relationship between SPIN1 and SPL11 [22]. In this study, we also found that the expression of *Spl11* is repressed in the *Rbs1* overexpression plants. In contrast, both the expression of *Rbs1* and accumulation of the RBS1 protein are increased significantly in the *spl11* mutant, indicating a negative relationship between RBS1 and SPL11. However, the direct relationship between these two proteins remains unclear. Yeast-two hybrid assays using SPL11 as the bait and RBS1 as the prey or vice versa did not detect any interaction between them (Vega-Sanchez and Wang, unpublished results). It is plausible that RBS1 is one of the components in the SPL11 protein complex and the interaction between RBS1 and SPL11 requires the presence of another protein that interacts with both proteins, for example SPIN1.

Because RBS1 is a putative RNA binding protein and has RNA binding activity *in vitro*, it might be involved in RNA metabolism to regulate flowering time. In mammals, it has been recently shown that hnRNP-R binds to the 3′UTR of serotonin N-acetyltranferase and to the AU rich element of c-Fos mRNA to promote their degradation [32], [33]. Whether *Rbs1* is involved in mRNA turnover in rice needs to be determined in future studies. It is interesting to note that the putative ortholog of RBS1 in *Arabidopsis*, LIF2, is also involved in flowering time control [19]. However, at this point, it is unclear whether LIF2 and RBS1 are functional homologs. LIF2 seems to be part of protein complex that regulates gene expression via epigenetic mechanisms [19]. Whether RBS1 acts similarly to regulate flowering and other developmental pathways needs to be explored. Complementation studies of the *Arabidopsis lif2* loss-of-function mutants with the rice *Rbs1* gene should provide clues as to whether these pathways are conserved or not between the two species.

## Supporting Information

Figure S1 **Identification of** ***Rbs1*** **by yeast two-hybrid screen in a rice cDNA library using the full length** ***Spin1*** **as the bait.** Numbers 72 and 69-2 are independent yeast clones containing the pDBleu-*Spin1* and pPC86-*Rbs1* constructs. Positive clones were identified by an X-GAL assay.
(TIFF)

Figure S2 **Molecular analysis and flowering time of the** ***rbs1*** **mutant.** A. Structure of the T-DNA insertion. T-DNA was inserted into the fourth intron of Rbs1. Arrows indicate the primers used for analyzing the insertion site. LB and RB represent the left and right borders of T-DNA; B. PCR genotyping of the rbs1 mutant. DJ denotes Dongjin, 1 is a segregated wild type, 2 and 3 are homozygous plants and 4 is a heterozygous plant; C. Expression of Rbs1 for the plants in S3B; D. Flowering time of the rbs1 mutant under LD and SD conditions.
(TIFF)

Figure S3 **Western blot analysis of the protein level of RBS1 in the** ***Rbs1*** **overexpression and wild type plants.**
(TIFF)

Figure S4 **Expression analysis of** ***Rbs1*** **in the** ***Spin1*** **overexpression plants,** ***spl11*** **mutant plants and wild type Nipponbare (NPB) plants.** A. RT-PCR analysis of *Rbs1* and *Spin1* in the *Spin1* overexpression and RNAi transgenic plants; B. RT-PCR of *Rbs1* in the leaves of the wild type IR68 and *spl11* mutant plants at different developmental stages. For *spl11*, young (Y) without lesions, fully expanded (FE) with few lesions and old leaves (O) with many lesions were taken for RNA extraction; C. RT-PCR of *Rbs1* in leaves from 55 day-old plants of the wild type IR64 and *spl11* mutant. D. Western blot analysis of RBS1 in the *spl11* mutant and *Spl11* overexpression lines.
(TIFF)

Table S1 (DOCX)
