# Supplementary figures and images for: RBS1, an RNA Binding Protein, Interacts with SPIN1 and Is Involved in Flowering Time Control in Rice
Source: PLoS One. 2014 Jan 30;9(1):e87258. doi: 10.1371/journal.pone.0087258 (PMC3907535; doi:10.1371/journal.pone.0087258)

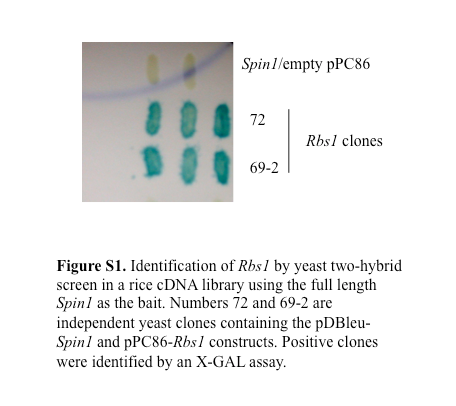

Supplement: Figure S1 — Identification of Rbs1 by yeast two-hybrid screen in a rice cDNA library using the full length Spin1 as the bait. Numbers 72 and 69-2 are independent yeast clones containing the pDBleu-Spin1 and pPC86-Rbs1 constructs. Positive clones were identified by an X-GAL assay. (TIFF) [file pone.0087258.s001.tiff]

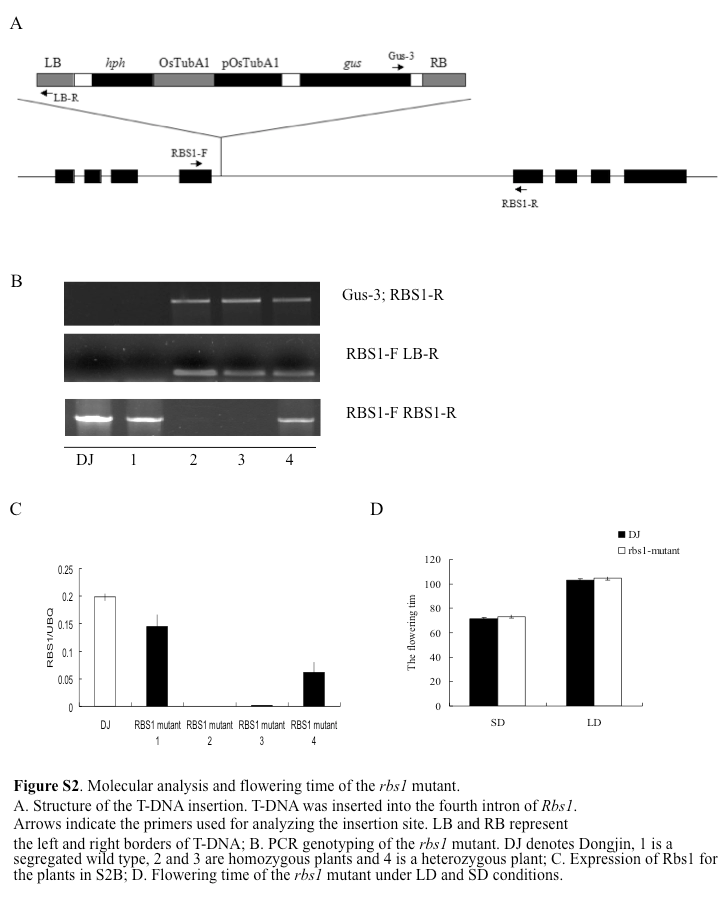

Supplement: Figure S2 — Molecular analysis and flowering time of the rbs1 mutant. A. Structure of the T-DNA insertion. T-DNA was inserted into the fourth intron of Rbs1. Arrows indicate the primers used for analyzing the insertion site. LB and RB represent the left and right borders of T-DNA; B. PCR genotyping of the rbs1 mutant. DJ denotes Dongjin, 1 is a segregated wild type, 2 and 3 are homozygous plants and 4 is a heterozygous plant; C. Expression of Rbs1 for the plants in S3B; D. Flowering time of the rbs1 mutant under LD and SD conditions. (TIFF) [file pone.0087258.s002.tiff]

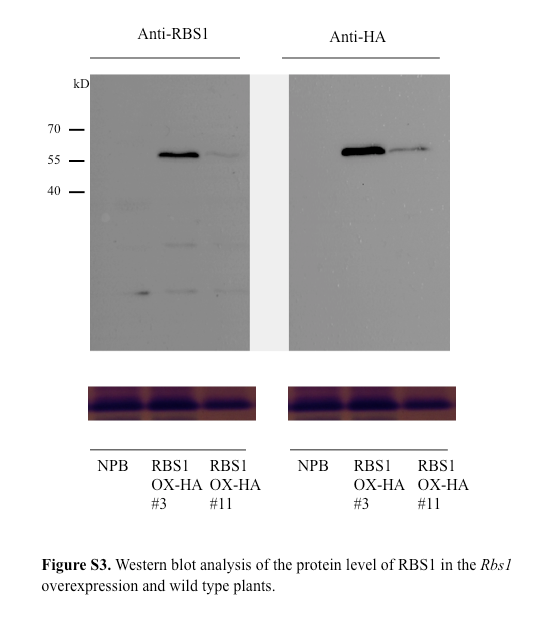

Supplement: Figure S3 — Western blot analysis of the protein level of RBS1 in the Rbs1 overexpression and wild type plants. (TIFF) [file pone.0087258.s003.tiff]

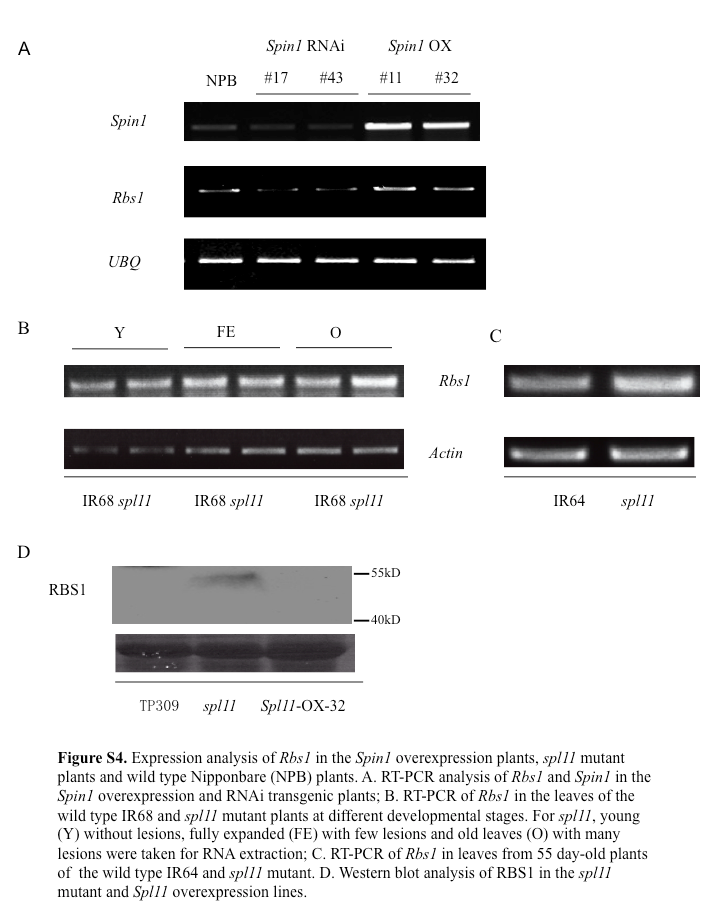

Supplement: Figure S4 — Expression analysis of Rbs1 in the Spin1 overexpression plants, spl11 mutant plants and wild type Nipponbare (NPB) plants. A. RT-PCR analysis of Rbs1 and Spin1 in the Spin1 overexpression and RNAi transgenic plants; B. RT-PCR of Rbs1 in the leaves of the wild type IR68 and spl11 mutant plants at different developmental stages. For spl11, young (Y) without lesions, fully expanded (FE) with few lesions and old leaves (O) with many lesions were taken for RNA extraction; C. RT-PCR of Rbs1 in leaves from 55 day-old plants of the wild type IR64 and spl11 mutant. D. Western blot analysis of RBS1 in the spl11 mutant and Spl11 overexpression lines. (TIFF) [file pone.0087258.s004.tiff]
