# Supplementary material for: RBS1, an RNA Binding Protein, Interacts with SPIN1 and Is Involved in Flowering Time Control in Rice
Source: PLoS One. 2014 Jan 30;9(1):e87258. doi: 10.1371/journal.pone.0087258 (PMC3907535; doi:10.1371/journal.pone.0087258)
Supplement: Table S1 — (DOCX) [file pone.0087258.s005.docx]

**Table S1. Primers and antibodies used in this study**

| **Name Sequence (5’-3’)** |
| --- |
| SPL11-QF TTTGAACGGCACCGATAGG  SPL11-QR GAGGCTTGCGACTGGGACT  Spin1-QF GGTATCCAGGTGCCTTATG  Spin1-QR GGACGATCTGCGACTATTG  RBS1-QF TTTCCGTTCCCCTGTGCCG  RBS1-QR ACACGACTATAATTGGAGAAA  RBS1XhoI F GCGCTCGAGATGTCCGACCGGCAGCAGTC  RBS1 SmaI R GAGCCCGGGGTAAGGGCGATATCGCCG  UbqRT F CGCAAGAAGAAGTGTGGTCA  UbqRT R GGGAGATAACAACGGAAGCA  Hd1-F GTT TGC AGA GAA GGA AGG GAG CGA GTG  Hd1-R GGT CGT GCC TCT GCA TAC GCC TTT CTT G  Ehd1 F TGG TTT GCT CAT TCT GCA AG  Ehd1 R TTC TAG AGG CGC TAG CGA AG  Hd3a-1-R TCAGGGTTTTTTGCAAGATCGATGG  Hd3a-1-F TGGGCTTGGTGCATCTGGGTCTACC  RBS1 F TTATAACCACGCTTGTGCAGAC  RBS1 R CTCTTATGCCCATCCTTAGCAG  RBS1 Antibody VDLEGDDDNDVMDDC  SPIN1 Antibody NPRSNRSGNGFSPW |
